# Neuropsychological assessment in adolescents with anorexia nervosa – exploring the relationship between self-report and performance-based testing

**DOI:** 10.1186/s40337-015-0062-2

**Published:** 2015-08-13

**Authors:** Kristin Stedal, Camilla Lindvall Dahlgren

**Affiliations:** Regional Department for Eating Disorders, Division of Mental Health and Addiction, Oslo University Hospital, Postboks 4956 Nydalen, 0424 Oslo, Norway

**Keywords:** Anorexia nervosa, Eating disorders, Neuropsychological assessment, Ecological validity

## Abstract

**Objective:**

Research investigating the relationship between neuropsychological test performances and self-reported cognitive functioning in patients with anorexia nervosa (AN) is limited, and existing experimental studies only demonstrate a low-to-moderate relationship between the performance based tests and everyday behaviour. The objective of the current study was to explore the association between neuropsychological test performance and self-reports of executive functioning in adolescents with AN.

**Method:**

Twenty adolescent females diagnosed with AN, aged 13 to 18, completed neuropsychological test battery “the Ravello Profile” and the self-report version of the Behaviour Rating Inventory of Executive Function (BRIEF-SR). The BRIEF Parent Form (BRIEF-PF) was employed to provide additional information of the patients’ executive functioning.

**Results:**

Based on group level analyses, the results support the existing literature in failing to find consistent weaknesses in neuropsychological functioning in adolescents with AN. Further, with few exceptions, the Ravello Profile was insubstantially correlated with the majority of the BRIEF clinical scales, indicative of a lack of association between these two assessment methods.

**Conclusion:**

The current study accentuates the need for concern regarding the generalizability of neuropsychological assessments in adolescent patients with AN.

## Background

Clinicians working in the field of eating disorders will often describe patients with anorexia nervosa (AN) as being preoccupied with details, and displaying high levels of cognitive and behavioural rigidity. This pertains in particular to issues concerning food, weight and bodily appearance, but also to other aspects of everyday functioning like schoolwork and in relation to friends and family. In adults with AN, neuropsychological assessments and clinical observations have led researchers to hypothesise that there could be an underlying brain based abnormality causing this characteristic cognitive profile [1–5], and that such abnormalities could represent an AN endophenotype rather than temporary, state dependent cognitive functioning [6–12]. In adolescents with AN, however, most research fails to find consistent neuropsychological weaknesses [13–16] and two recent reviews on set-shifting and central coherence support the notions that such difficulties are less pronounced in this patient group [17, 18].

Several hypotheses have been put forward to explain why the neurocognitive profile of young AN patients differs from that of adults. According to Bühren et al. [13, 19], set shifting in children and adolescents is a skill that has yet to evolve, and the brain maturation, especially the cortico-striatal networks which modulate set-shifting, are not fully developed until early thirties, and thus, cannot be labelled defective. Previous studies have also suggested weaknesses in neuropsychological functioning as being a consequence of the illness, and representing a scar effect with its severity depending on the duration of illness [19–21]. In line with this hypothesis, it is possible that, although many young patients with AN do not exhibit neuropsychological difficulties, such will emerge as the illness progress, and hence, be detectable as the course of the illness extends. It could also be argued that the instruments used to assess neuropsychological functioning fail to capture cognitive challenges as they appear in contextually meaningful settings. Clinically, many young individuals with AN undoubtedly struggle with being flexible and being able to see “the bigger picture”, especially in terms of eating disorder (ED) specific symptoms such as weight, shape, food and purging behaviours. However, as these weaknesses do not always appear to manifest in a controlled test-setting, this might indicate low sensitivity of assessment measures, that is, that they are not sensitive enough to pick up on modest neuropsychological weaknesses, or perhaps even more realistic, that rigidity and detail focus related to everyday life activities are non-assessable using neuropsychological tests, i.e., the tests lack ecological validity.

Neuropsychological assessments were historically employed as a way of localising and detecting neuropathology. Based on an individual's cognitive profile of strengths and weaknesses, inferences would be drawn regarding brain dysfunctions and the probable aetiology [22]. It is frequently assumed that results from neuropsychological performance tests can predict every day functioning [23], and it is commonly expected that poor performance on a neuropsychological test will be of relevance for clinical purposes and in terms of the patient’s day-to-day functioning. Surprisingly, there has been very little research investigating the accuracy of this assumption, and existing experimental studies recurrently demonstrate only a weak-to-moderate relationship between the performance on neuropsychological tests and everyday behaviour [24–27]. Further, performance-based tests of executive functioning have been suggested to be more sensitive to deficits in adults than in children, as they were originally developed for use with adult populations [28].

In 2004 a neuropsychological test battery named “the Ravello Profile” was developed as an assessment tool specifically aimed at investigating cognitive functioning, and in particular, various aspects of executive functioning in patients with AN [29]. Several studies employing this test battery in AN populations have been published [16, 30–32], but the results so far have only demonstrated small to insignificant impairments in adolescents with AN [16, 30–32]. This is in opposition to the previous literature on cognitive functioning in adults with AN, but perhaps even more pertinent, in complete contrast to how clinicians, parents and teachers describe these patients in terms of rigidity and preoccupation with detail, order and symmetry. As quantifying everyday cognitive functioning is a complex task, and as any form of assessment is associated with a certain degree of error [33], one might be inclined to ask oneself how to most accurately assess neuropsychological function in adolescents with AN. Recently, novel measures assessing executive functions in contextually relevant settings have emerged with the aim of complementing test-based results and increase ecological validity. The Behavior Rating Inventory of Executive Function (BRIEF) [34] was developed as a tool for assessing executive functioning in children and adolescents in an ecologically valid manner. It yields evidence of executive function behaviours in school and home environments, and can serve as an important adjunct to clinical evaluation and neuropsychological assessments of executive dysfunction [35]. The BRIEF has been extensively employed in assessing executive functions in attention deficit hyperactivity disorder (ADHD) [36–38] and in traumatic brain injury research [39, 40], but its use in research on eating disorders has so far been limited [41, 42].

The aim of the current study was to explore the relationship between performance based- and self-reported neuropsychological functioning in adolescents with AN. The means through which this was achieved, was by comparing tests results derived from the Ravello Profile subtests, and self- and parent reports of executive functioning assessed using the BRIEF-SR [43] and BRIEF-PF [34, 44].

## Method

### Participants

Data were collected as part of a feasibility trial of cognitive remediation therapy (CRT) for adolescents with AN [16, 41]. The sample consisted of 20 female in- and outpatients currently in treatment for AN. Patients were between 13 and 18 years of age (Mean = 15.9, *SD* = 1.6) and ethnic Norwegian. Ten of the patients were recruited from a paediatric outpatient service, and the remaining ten from a regional inpatient service. At time of assessment, inpatients were hospitalized and receiving high-intensity physical and psychological treatment. The outpatients were engaged in less intense treatment, primarily oriented towards physical aspects of the illness. The Regional Committee for Medical Research Ethics (REK) granted ethical approval to conduct the study. All patients were fully informed about the study, and gave written informed consent. Parental consent was obtained for patients below the age of 16.

There were no significant differences between in- and outpatients on any of the baseline assessment variables. Patients’ weight and height was measured in conjunction with assessments, with results revealing a mean BMI percentile just above 10 % (Mean = 10.2, *SD* = 17.2). The Eating Disorder Examination Questionnaire version 6.0 (EDE-Q 6.0) [45] was used to assess eating disorder psychopathology, with results yielding a mean global EDE-Q score of 3.4 (*SD* = 1.4). Based on the results from the EDE-Q assessing binge eating and compensatory behaviours, 18 patients were classified as having a restricting subtype of AN (AN-R), and the two remaining patients fitted the description for a binge-purge subtype (AN-BP). Verbal intelligence was assessed using the Vocabulary subtest from the Wechsler Adult Intelligence Scale – Third Edition (WAIS-III) [46], and the Wechsler Intelligence Scale for Children – Third Edition (WISC-III) [47]. Performance-IQ was assessed using the Matrix Reasoning subtest from the WAIS-III, and the Wechsler Abbreviated Scale of Intelligence (WASI) [48]. All patients scored within the normal range on the measures assessing intelligence.

## Assessment

### Self-report measures

#### BRIEF-SR

The self-report version of the Behavior Rating Inventory of Executive Function (BRIEF-SR) [43] yields information about young people’s views of their own executive functions (EF) in every day life settings, and is designed for children and adolescents between the ages of 11 and 18. The 80-item questionnaire is scored on a 3-point Likert scale: “never”, “sometimes”, and “always,” and provides scores on eight clinical scales describing various aspects of EF. Four subscales, Inhibit, Shift, Emotional Control and Monitor, are combined to produce a broader index called the Behavior Regulation Index (BRI). The four remaining subscales, Working memory, Plan/Organize, Organization of materials and Task completion, comprise the Metacognitive Index (MI). The Global Executive Composite (GEC) is the overall score, which is a composite of the two index scores. Scaled BRIEF-SR scores are transformed to age and gender corrected t-scores (M = 50, SD =10), for which higher scores indicate higher degrees of executive dysfunction. T-scores at or above 65 (i.e. 1.5 SD above the normative mean) are considered clinically significant in terms of executive dysfunction.

#### BRIEF-PF

The BRIEF Parent Form (BRIEF-PF) [34, 44] is to be completed by the caregiver, and is an 86-item questionnaire designed to provide detailed knowledge of the child’s behaviour. It overlaps with the BRIEF-SR on the following subscales: Inhibit, Shift, Emotional control, Working memory, Plan/Organize, Organization of materials and Monitor. Adding a ninth subscale labelled “Initiation”, these clinical subscales together produce the same two indexes as the BRIEF-SR: the BRI and the MI, as well as a composite score (GEC). Scoring procedures are identical for the BRIEF-PF and BRIEF-SR. The BRIEF-SR and BRIEF-PF clinical scales are described in more detail in Table 1.

**Table 1:** BRIEF-SR & BRIEF-PF clinical scales

Clinical scale	Description and intrpretations of scale
Inhibit	The ability to inhibit, resist or not act on an impulse. Also refers to the ability to stop one’s own behavior at an appopriate time.
Shift	The content-dependent ability to move freely from one activity, situation or aspect of a problem to another. Includes making swift transitions, problem-solve flexibly and switch or alternate attention.
Behavioral shift^1^	To be able to flexibly alter your behavior depending on environment or schedule
Cognitive shift^1^	To flexibly solve problems
Emotional control^1^	The ability to adjust emotional responses in an appropriate and constructive way
Monitor	The ability of the child to assess its own performance to attain knowledge of progress in terms of personal goals and achievements. Also a personal monitoring functioning to ensure the effect on one’s own behaviors on others
Working memory	The ability to hold information in mind with the purpose of completing a task or activity
Plan/Organize	The ability to tackel demands, both current and future-oriented. To be able to anticipate future demands, to set goals and to time-efficiently develop strategies for goal achievement
Organization of materials	The orderliness of work, play and storage spaces (bedrooms, lockers, desks etc.)
Task completion^1^	The ability to finish or complete task appropriately and within a given timeframe
Initiate^2^	The ability of initiation an assignment or activity. To be able to independently generate ideas.

^1^= Only in BRIEF-SR

^2^= Only in BRIEF-PF

Both BRIEF-SR and BRIEF-PF are standardized assessment instruments with well-established psychometric properties [43]. For the BRIEF-SR, high internal consistency has been shown for the 80-item GEC (.96), and test-retest reliability is supported by correlations among clinical scales ranging from .59 to .85. Higher correlations were observed for the index scores BRI and MI (.84 and .87 respectively), with the highest correlation (.89) emerging for the GEC [49]. Convergent evidence of validity is further supported by moderate correlations (for GEC, *r* = .56) between BRIEF-SR and BRIEF-PF. For the BRIEF-PF, high internal consistency has been reported (.80 - .98), and test-retest correlation ranging from .81 (clinical scales) to .88 (GEC) with the highest correlation emerging for the metacognitive index (MI) (.88) [34]. Further, preliminary analyses have supported its reliability and validity in assessing EF in adults with AN [50]

#### Neuropsychological Assessment

The neuropsychological test battery “the Ravello Profile” [29, 51] (Table 2) was developed specifically to assess individuals with AN, and is suitable for individuals between the ages of 8–89. The test battery includes a variation of subtest from the Delis Kaplan Executive Functioning System (D-KEFS) [52], the Rey Complex Figure Test (RCFT) [53, 54], and the Brixton Test [55]. For more detailed description and information regarding the Ravello Profile, see Rose et al. [29, 51].

**Table 2:** The Ravello Profile

Domain	Task	Assesment material
IQ		
Performance IQ	Matrix Reasoning	WAIS-III / WASI
Verbal IQ	Vocabolary	WAIS-III / WASI-III
Executive Functioning		
Cognitive Inhibition	Colour Word Interference Condition 3	D-KEFS
Cognitive Inhibition & Flexibility	Colour Word Interference Condition 4	D-KEFS
Verbal Fluency	Verbal Fluency Condition 1	D-KEFS
Verbal Fluency	Verbal Fluency Condition 2	D-KEFS
Switching	Verbal Fluency Condition 3	D-KEFS
Switching	Trial Making Test Condition 4	D-KEFS
Planning & Inhibition	Tower of London	D-KEFS
Flexibility & Spatial Working	Brixton Spatial Anticipation Test	Hayling & Brixton
Memory		
Visio-Spatial Memory	Immediate Recall	RCFT
	Delayed Recall	RCFT
Visual Spatial Processing	RCFT Style Index	RCFT
	RCFT Order of Construction Index	RCFT
	RCFT Central Coherence Index	RCFT

*WAIS-III* = Wechsler Adult Intelligence Scale – Third Edition (47), *WASI* = Wechsler Abbreviated Scale of Intelligence – Third Edition (49), *WISC* = Wechsler Intelligence Scale for Children – Third Edition (48), *D-KEFS* = Delis Kaplan Execution Function System (52), Hayling & Brixton (55), *RCFT* = Rey Complex Figure Test (53,54)

### Procedure

Patients and parents completed the BRIEF-SR and BRIEF-PF questionnaires in conjunction with the patients’ neuropsychological assessment (i.e. the Ravello Profile), which were administered by two trained investigators. A total of 14 mothers and 6 fathers completed the BRIEF-PF.

#### Statistical Analyses

All statistical analyses were carried out using PASW^©^ Statistics 18 for Windows XP/Vista®. For comparative purposes, the neuropsychological raw scores were converted to *z*-scores using means and *SD*s using the tests’ published age-based norms. Z-scores have a mean of zero and a standard deviation of 1. Exceptions were the Brixton Spatial Anticipation Test and the Central Coherence measure for which norms for children and adolescents are not available. Consequently, control group means from a recently published study employing these measures in a child and adolescent sample were used (N =66) [31]. The relationship between neuropsychological performance (as measured by the tests in the Ravello Profile) and self-reports of executive functioning (as measured by the BRIEF-SR and BRIEF-PF scales) was investigated using the Spearman rank order correlations rho. Rho (*ρ)* is defined as *small* (.10 to .29), *medium* (.30 to .49) or *large* (.50 to 1.0). Due to the exceptionally large number of correlations (N = 384), we chose to focus on the magnitude of correlations rather than their statistical significance. Only large correlations (.50 ≥) were considered being of interest.

## Results

### Neuropsychological functioning, BREIF-SR & BRIEF-PF scores

Table 3 present *z*-scores on the Ravello Profile subtests, and on the BRIEF-SR & BRIEF-PF subscales. As evident from the table, all neuropsychological test scores fell within the normal range. This was also true for both BRIEF-SR and BRIEF-PF scores (correspondent *t*-scores have been presented elsewhere [41]).

**Table 3:** The Ravello Profile, BRIEF-SR and BRIEF PF z-scores (N = 20)

ᅟ												
ᅟ	ᅟ	ᅟ	ᅟ	ᅟ	ᅟ	ᅟ	ᅟ	ᅟ	ᅟ	ᅟ	ᅟ	
ᅟ	ᅟ	ᅟ							ᅟ	ᅟ	ᅟ	
Ravello Profile mean z-scores (*SD*) - *patients*												
RCFT	RCFT	RCFT	Verbfl3	TMT4	Stroop3	Stroop4	Tower	Brixton	RCFT	RCFT	RCFT	
Immediate	Delayed	Recogn.							Style	Order	CCI	
-.61 (1.23)	-.69 (1.38)	-.37 (1.35)	.68 (.90)	-.65 (.76)	-.32 (1.15)	.00 (86)	.42 (.71)	.12 (1.28)	.55 (.7)	.48 (1.02)	.64 (.79)	
BRIEF-SR mean z-scores (*SD*) - *patients*												
		Behavioral	Cognitive	Emotional	Working	Plan/	Org. of	Task				
Inhibit	Shift	shift	shift	control	Memory	Organise	material	completion	Monitor	BRI	MI	GEC
-.06 (1.34)	1.44 (1.39)	1.42 (1.63)	.93 (1.28)	1.23 (1.22)	.19 (1.37)	.03 (1.16)	-.39 (1.07)	.57 (1.60)	-.16 (1.02)	.83 (1.32)	.08 (1.46)	.54 (1.42)
BRIEF-PF mean z scores (*SD*) - *parents*												
			Emotional	Working	Plan/	Organization of						
Inhibit	Shift	Initiate	control	memory	organise	materials	Monitor	BRI	MI		GEC	
.12 (1.12)	1.20 (1.08)	.21 (1.01)	1.52 (1.12)	.46 (1.28)	.33 (1.06)	-.77 (.80)	.18 (.79)	1.11 (1.03)	.18 (.93)		.55 (.89)	

*SD =* Standard Deviation, *BRIEF SR =* Behavioral Rating Inventory of Executive Funtion –Self Report, *BRIEF-PF* = Behavioral Rating Inventory of Executive Funtion – Parent Form, *RCFT* = Rey Complex Figure Test, *Verbfl* = Verbal Fluency, *TMT* = Trial Making Test, *Style* = Style index, Order = Order of construction index, *CCI* = Central coherence index, *BRI* = Behavioral Regulation Index, *MI* = Metacognitive Index, *GEC* = Global Executive Composite

### Correlational analysis

Negative medium correlations were observed for the Tower task and five BRIEF-SR subscales. The results also revealed a large positive correlation between the RCFT, OCI and the BRIEF-SR scale Task completion, and medium correlations between scores on WAIS/WISC Vocabulary and two BRIEF-SR subscales. As for correlations between parents’ reports of their children’s executive functions (BRIEF-PF) and the children’s neuropsychological test performance, the correlational analysis revealed large negative correlations between the TMT and two BRIEF-PF subscales, a large negative correlation between the Tower task and the BRIEF-PF inhibit subscale, as well as medium to large negative correlations between WAIS/WISC Vocabulary and a number of subscale and composite BRIEF-PF scales. Details are presented in Table 4 and 5.

**Table 4:** Spearman rank order correlations (*ρ*) between the Ravello Profile tests and the BRIEF-SR scales (N=20)

*ᅟ*														
		ᅟ	ᅟ	ᅟ	ᅟ	ᅟ	ᅟ	ᅟ	ᅟ	ᅟ	ᅟ	ᅟ	ᅟ	ᅟ
BRIEF-SR														
		Inhibit	Shift	Emotional control	Monitor	Working memory	Plan/Organise	Organisation of materials	Task completion	BRI	MI	GEC	Behavioral Shift	Cognitive shift
The Ravello Profile	RCFT Immediate	.19	-.01	-.07	.08	.06	.36	.14	.30	.07	.18	.25	.03	-.10
	RCFT Delayed	.17	.03	.02	.08	.01	.40	.09	.29	.10	.15	.23	.10	-.05
	RCFT Recognion	.07	.09	.06	.03	-.09	.10	-.12	-.05	.10	-.06	-.02	.07	.11
	RCFT Style	-.07	.02	-.17	-.12	-.06	.24	-.10	.25	-.09	.05	.07	.03	.01
	RCFT Order	.10	.04	.05	.11	.36	.49	.23	**.50**	.06	.46	.34	-.04	.16
	RCFT CCI	.22	.12	.02	.14	.25	.39	.31	.36	.12	.35	.35	.02	.22
	Verbal Fluency 1	.26	.14	.10	-.07	.04	-.02	-.01	-.35	.14	-.20	.08	.12	.08
	Verbal Fluency 2	.10	.04	.23	.03	-.20	-.09	-.08	-.26	.13	-.20	-.04	.22	-.16
	Verbal Fluency 3	-.00	-.16	-.11	-.07	.05	.09	-.23	.05	-.10	-.18	-.02	-.14	-.23
	Trail Making Test 4	.24	-.19	.11	-.00	.00	-.04	-.06	-.41	.15	-.18	.06	-.15	.01
	Stroop 3	.49	.16	.37	.36	.11	.10	.16	-.16	.44	-.00	.20	.04	.08
	Stroop 4	.37	.31	.18	.08	-.06	.07	.06	-.16	.36	-.25	.19	.17	.18
	Tower	-.27	-.48	-.45	-.27	-.26	**-.53**	-.16	-.19	-.45	-.22	-.48	**-.50**	-.39
	Brixton	-.16	-.22	-.29	-.10	-.09	.05	-.30	-.07	-.23	-.20	-.06	-.07	-.36
	WAIS/WASI Matrix	.44	-.17	.09	.21	.09	-.04	-.03	-.26	.19	-.05	.07	-.16	-.19
	WAIS/WISC Vocabulary	-.10	-.03	-.19	-.33	-.15	-.02	-.20	-.20	-.11	-.33	-.10	-.21	.07

All correlations are based on *z*-scores. Spearman’s rank order correlations (*ρ*) are defined as *small* (.10 to .29), *medium* (.30 to .49) or *large* (.50 to 1.0). Large correlations (.50 ≥) are marked in bold.

*BRIEF-SR* = Behavior Rating Inventory of Executive Function – Self-Report, *RCFT* = Rey Complex Figure Test, *Immediate* = Immediate recall, *Delayed* = Delayed recall, *Style* = Style index, *Order* = Order of construction index, *CCI* = Central coherence index, *WAIS* = Wechsler Adult Intelligence Scale, *WASI* = Wechsler Abbreviated Scale of Intelligence, *WISC* = Wechsler Intelligence Scale for Children, *BRI* = Behavior Regulation Index, *MI* = Metacognitive Index, *GCE* = Global Executive Composite

**Table 5:** Spearman rank order correlations (*ρ*) between the Ravello Profile tests and the BRIEF-PF scales (N=20)

ᅟ												
		Inhibit	ᅟ	ᅟ	Initiate	ᅟ	ᅟ	ᅟ	ᅟ	ᅟ	ᅟ	ᅟ
BRIEF-PF												
		Inhibit	Shift	Emotional control	Initiate	Working memory	Plan/organise	Organisation of materials	Monitor	BRI	MI	GEC
The Ravello Profile	RCFT Immediate	-.10	.25	.08	.08	.09	.17	-.07	.15	.11	.11	.13
	RCFT Delayed	-.08	.19	.01	.17	.00	.12	.03	.11	.03	.12	.10
	RCFT Recognion	.13	.10	.32	.11	-.18	-.12	.25	-.02	.17	-.02	.02
	RCFT Style	-.25	.14	.01	.27	.10	.19	-.02	-.14	.03	.18	.15
	RCFT Order	-.25	.16	-.17	.20	.21	.29	.27	.02	-.13	.28	.14
	RCFT CCI	-.27	.04	.20	.19	.20	.21	.32	-.01	-.12	.26	.13
	Verbal Fluency 1	.37	-.10	.06	-.20	-.15	-.03	-.13	.16	.15	-.07	.00
	Verbal Fluency 2	.31	.12	.23	.04	-.08	-.07	-.31	.36	.20	-.01	.07
	Verbal Fluency 3	.30	.06	.10	-.26	-.30	-.13	-.44	-.04	.28	-.27	-.08
	Trail Making Test 4	-.02	-.37	-.22	-.45	**-.63**	-.43	-.19	-.14	-.24	**-.51**	-.47
	Stroop 3	-.17	-.23	-.11	-.26	-.48	-.40	-.25	-.14	-.23	-.42	-.42
	Stroop 4	.04	-.28	-.08	-.26	-.39	-.15	-.32	-.24	-.05	-.35	-.27
	Tower	**-.57**	-.08	-.20	-.09	.04	-.06	.09	-.36	-.27	-.09	-.14
	Brixton	.30	.21	.19	-.12	.04	.19	-.33	-.04	.40	-.01	.17
	WAIS/WASI Matrix	.21	.25	.35	-.08	-.22	-.26	-.03	.02	.33	-.21	.01
	WAIS/WISC Vocabulary	-.22	-.38	-.21	-.45	**-.56**	-.19	-.19	-.35	-.31	-.45	-.44

All correlations are based on *z*-scores. Spearman’s rank order correlations (*ρ*) are defined as *small* (.10 to .29), *medium* (.30 to .49) or *large* (.50 to 1.0). Large correlations (.50 ≥) are marked in bold.

*BRIEF-PF* = Behavior Rating Inventory of Executive Function – Parent Form, *RCFT* = Rey Complex Figure Test, *Immediate* = Immediate recall, *Delayed* = Delayed recall, *Style* = Style index, *Order* = Order of construction index, *CCI* = Central coherence index, *WAIS* = Wechsler Adult Intelligence Scale; *WASI* = Wechsler Abbreviated Scale of Intelligence, *WISC* = Wechsler Intelligence Scale for Children, BRI = Behavior Regulation Index, *MI* = Metacognitive Index; GCE = Global Executive Composite

## Discussion

To the authors knowledge, this is the first study investigating the relationship between a neuropsychological assessment method specifically aimed at assessing patients with AN (i.e. the Ravello Profile) and self-reports of executive functioning. The study highlights the fact that the conceptualizations behind these test were to aid in the diagnosis of neuropathology – not as means of predictions about the functioning of psychiatric populations in real-life or treatment settings [56]. The results are in line with previous studies demonstrating only a low-to-moderate relationship between reports of everyday skills and scores on neuropsychological tests [57–59], and support the existing literature in failing to find consistent weaknesses in neuropsychological functioning in adolescents with AN. The results further accentuate the need for concern regarding the generalizability of neuropsychological assessments in adolescent patients with AN.

On an overall level, there was a lack of correlations between the tests in the Ravello Profile and the BRIEF-SR and BRIEF-PF clinical subscales. This lack of correlations could be attributed to a variety of interpretations. Firstly, it is possible that the two assessment methods simply measure different types of executive functions. Secondly, there is a risk that BRIEF reports might be biased due to the nature of patient care for half of the patients (i.e. inpatients) making it difficult for patients and parents to assess “normal” day-to-day behaviour. Further, as the two assessment methods vary greatly, it is not unlikely that factors such as personality, environmental influence and personal efforts had an uneven impact on performance based and self-reported scores.

However, there were some exceptions. The results demonstrate an association between the WAIS/WISC vocabulary subtest and several of the BRIEF-SR and BRIEF-PF subscales. These findings are comparable to those of Vriezen and Pigott [28] who investigated the relationship between the parental report of the BRIEF and performance based measures of executive function in children with moderate to severe traumatic brain injury. Results from this study revealed that the BRIEF did not correlate with any of the traditional performance-based tests of executive function (the Trail making test B, the Verbal fluency test and the Wisconsin card sorting test), but that verbal intelligence as measured by the WISC-III correlated with metacognitive aspects of executive functioning such as initiation, organisation and monitoring of activities [28]. The Vocabulary subtest of the WAIS/WISC has previously been shown to be closely related to overall verbal intelligence [60, 61]. The current study yields support to the strong association between verbal abilities and the capability to monitor and assess performance, and to acquire knowledge in terms of personal goals and achievements.

Further, it is of interest to note that the Tower test was correlated with a number of the BRIEF-SR scales such as Shift, Behavioral shift, Emotional control and Plan/Organize. This is in line with previous studies suggesting that the Tower test is a complex task requiring multiple aspects of executive functioning, including planning [62, 63], inhibition [64] and working memory [65, 66]. In the current study, Tower test performance was related to the ability to flexibly shift and alternate attention, to track demands - both current and future oriented - as well as to keep emotional control during a rather stressful and demanding task. The large correlation between the Tower test and these BRIEF-SR subscales also indicates that the Tower test might be a neuropsychological performance test which is highly applicable for assessing executive functioning in patients with AN. The Tower test measures several aspects of functioning, and appears to be a more ecologically valid instrument compared to some of the other tasks included in the Ravello Profile. This is in line with previous studies demonstrating that tests, which assess several executive domains simultaneously, are more similar to life-like challenges [67].

The majority of tests used in studies assessing neuropsychological functioning in patients with AN are instruments with a long history within the field of neuropsychology. The choice of specific tests is often based on its professed ability to assess specific cognitive domains, for example executive functioning. However, due to the fact that the majority of such tests have an inherent complexity, few of them actually measure merely a single function or ability. Also, poor performance on individual tasks could be the result for a variety of sub-optimal functioning skills [22], rather than a specific weakness. The results from studies investigating neurocognitive functioning in children and adolescents with AN have been highly inconsistent, and it is probable that the variability in results could be due to performance-based tests of executive functioning being more sensitive to deficits in adults than in children [28]. Further, albeit being sensitive in discriminating participants with a brain injury from controls [22], neuropsychological tests might not be particularly effective when it comes to predicting every day difficulties. Thus, test results within the normal range do not necessarily imply evidence of absence of abnormal brain functioning [68]. In addition, traditional neuropsychological tests do not take into account factors like; personality, the support from family and surroundings, as well as performance motivation, which can make them less able to predict real-life performance [68].

Because of the complex nature of executive functioning there are many difficulties associated with the assessment of these skills - perhaps in particular when attempting to say something about its relevance to cognitions and behaviours relevant to the everyday life of patients [23, 69, 70]. A neuropsychological test performance falling within the norm could mask a greater effort required by a person suffering from sub-optimal brain functioning. Studies have demonstrated that children can perform well on standardized neuropsychological tests whilst still having difficulties with everyday activities demanding strong executive functioning capacities [71]. Neuropsychological assessments are usually conducted free of distractions, in a quiet room with a test administrator who will carefully explain the rules, set goals and initiate and stop behaviours where appropriately [72]. This means that the testing environment itself can ameliorate difficulties with executive functions like starting and stopping behaviours, completing tasks and staying focused on the test being presented. Thus, a possible interpretation of the findings on the Ravello Profile in this study is that the structured setting of the neuropsychological assessment situation aids the patients performance, whilst the BRIEF provides an assessment of executive functioning which more accurately represents true day-to-day day functioning.

To the authors knowledge this is the first study to explore the relationship between Ravello Profile subtests and self-reports of executive functioning in adolescents with AN. However, the findings should be treated by caution as they are limited by a small sample size and the potential of truncated distribution of scores. Future studies should increase the number of participants to strengthen the validity and generalizability of results. The use of a self-report measure is a further limitation when interpreting the findings from this study. Illness and self-awareness is closely related to executive functions, and there is a tendency for patients with executive functioning difficulties to underestimate their own problems [73], thus the self-report version of the BRIEF (BRIEF-SR) might alone not be adequate for assessing impaired executive functioning in young patients with AN. Rather, by supplementing the BRIEF-SR with the report of relatives and/or teachers, the participants difficulties might be more accurately determined. Discrepancies between BRIEF-SR and BRIEF-PF ratings in the current population have been previously described by Dahlgren et al. [41] supporting the need for informant ratings of executive functioning in adolescents with AN. The Ravello Profile, the BRIEF-SR and the BRIEF-PF scores were all below the clinical cut-off level when analysed by group mean scores. This highlights the notion that, although these patients are commonly described as displaying executive dysfunctional behaviours and rigid cognitions, the scores on both the performance based tasks and behaviour rating inventories are within the normal range. As the BRIEF was developed specifically to yield ecologically valid measures of executive functions, one would expect it to be fairly sensitive to such difficulties in day-to-day functioning [44]. However, it has been demonstrated that there is great variability in scores on neuropsychological tests of executive functioning in patients with AN, and that individuals who struggle with these tasks can be masked when performing whole group analyses [30]. Future studies of executive functioning in adolescent patients with AN should aim at emphasizing assessments more closely related to real life, and to place grater focus on observations of behaviours of structured tasks performed in real world settings. Naturalistic assessment procedures, like the Multiple Errands Test [74], is an example of an approach where the aim is to obtain a greater understanding of the participants’ real world functioning. Some preliminary work on assessments of executive functions using virtual reality has also been proposed [75, 76], and could be helpful for improving ecological validity in assessing executive functions in this patient group. Combining performance-based tests with information from behavioural observations, interviews, informant ratings and self-report measures could enhance its relevance to everyday life. When employing a combination of neuropsychological performance tests and self-reports, difficulties experienced by patients can be further delineated, and psychological interventions can be better targeted. By taking a broad minded approach to neurocognitive assessments of young patients with AN, the possibility of better targeted interventions aimed at ameliorating cognitive challenges in the everyday life of the patients can be facilitated. Further, there is a lack of assessment tools targeting the evaluation of executive dysfunction in relation to ED specific cognitions and behaviours such as body shape and weight. At this point, patients with AN are often described as rigid and preoccupied with details, but researchers have yet to clarify whether such cognitive and behavioural characteristics pertains to the eating disorders per se, or if such categorizations reflects alterations in global executive function.

Finally, it is worth pointing out that the study holds some limitations with regards to the use of neuropsychological tests to reflect every day function. As mentioned previously, the tests used are based on a deficit model aiming to identify and quantify cognitive deficits, and might not be entirely suitable for the study of functional problems of people with anorexia nervosa scoring within the normal range. We can therefore not draw definite conclusions, and future studies are warranted to address this issue.

## Conclusions

The neuropsychological test battery, the Ravello Profile, was insubstantially correlated with the majority of the BRIEF clinical subscales, accentuating the need for concern regarding the generalizability of neuropsychological assessments in adolescent patients with AN. Future studies should aim to increase the sample size in order to be able to generalize findings, control for multiple comparisons and, potentially, look at executive functioning directly related to eating disorder psychopathology.
